# Supplementary material for: Interpreting polygenic score effects in sibling analysis
Source: PLoS One. 2024 Feb 15;19(2):e0282212. doi: 10.1371/journal.pone.0282212 (PMC10868763; doi:10.1371/journal.pone.0282212)
Supplement: Attachment — Submitted filename: Response to Reviewers 8.4.22.docx [file pone.0282212.s002.docx]

5. Review Comments to the Author

Please use the space provided to explain your answers to the questions above. You may also include additional comments for the author, including concerns about dual publication, research ethics, or publication ethics. (Please upload your review as an attachment if it exceeds 20,000 characters)

Reviewer #1: In this manuscript, the authors explored these expectations by using family (quad) data and simulations that include indirect genetic effect processes and evaluates the ability of sibling analysis to uncover direct genetic effects of polygenic scores. They pointed out that interpreting results from sibling analysis aimed at uncovering direct genetic effects should be treated with caution.
Overall, it is a solid manuscript. The authors conducted a variety of simulation scenarios to demonstrate the bias. The methodology and procedures were proposed and clearly described. The results and conclusions were clear and logical. The limitation is that the results are from a “simple” data generating processes, where they assumed no assortative mating, no gene-environment interaction or correlation, and sibling genetic correlations of exactly 0.5. But I understand that it is much more complicate in the real world.

My comments are:

1. the authors did 15 simulation repetitions, for me 15 times is kind low. I would expect that the results are generated from at least a few hundreds of repetitions.

*We thank the reviewer for raising this point. We have revised the figures to show the output of 200 simulations.*

2. In each of the boxplot, we can see that some groups have larger variations then others. Is there any explanation?

*We thank the reviewer for this comment. The updated figures show smaller variations across settings, suggesting that some of the variation in the figures from our original submission was due to the small number of simulations and the associated undue influence of a single simulation when only a small number are estimated.*

Minor comments:
1.The reference style is not consistent. For example, Introduction, second paragraph.

*We thank the reviewer for this comment and have harmonized our reference style. The second paragraph contains a footnote and also several references.*

2. The authors should fix all the math symbols and letters. They are not showed on the pdf file.

*We thank the reviewer for this comment and have checked to make sure the revised PDF properly displays the symbols/letters.*

Reviewer #2: The authors use simulated and real-world data (from parent-children quads), to determine whether within-family analyses using polygenic scores can truly separate direct and indirect genetic effects. This analysis seems especially important given that this is a claim that is often made, but not previously tested. The authors find that even under the ideal scenario (all direct, no indirect effects), within-family analyses tend to underestimate the true effect. As indirect and direct genetic effects become more correlated, estimate from within-family models become more biased in an unpredictable manner. This is an important investigation of a common assumption: that within family models are robust estimates of true direct genetic effects. It would be a welcome addition to the literature. My comments are mostly minor in hopes of clarifying some of the information provided in this manuscript.

Major points

Is this really a limitation of genetic analyses only? Could this be a broader issue of family fixed effects models in social and behavioral sciences more broadly? Is there any reason to think that the issues raised in these simulations do not also apply to phenotypic or environmental analyses within family?

*We thank the reviewer for the thoughtful question. While the broad issues we raise could potentially affect broader uses of within-family designs, we point out that genetic transmission is a special case and differs with environmental and phenotypic transmission. That is, within biological families, genetic variants are passed down directly to children in a way that differs from environments and phenotypes—there is a mechanical (biological/genetic) transmission that is ‘fixed’ in a way that environments and phenotype transmission is not fixed. If both parents have a zero minor allelles in a specific location of the genome, a child is assured of also having zero minor allelles at that same location (with the minor issue of de novo mutations excepted). There is no environment nor phenotype that has this type of “mechanical” transmission. The other side of this “mechanical” transmission is that the transmission is also permanent. The child who inherits zero minor allelles at a given location will always have the same zero minor alleles. This permanence is also not true for environments or phenotypes, which can change over development. We believe that additional, future work could extend our model to explore these broader classes of analyses but our paper is meant to make a more narrow point about the use of PGS/genetics in family based analyses.*

Minor points:

Moving the data and methods into the main text would be helpful. I know that it helped orient me to the analyses.

*We thank the reviewer for the comment. We follow the Plos One convention of separating the data and methods but do understand and sympathize with the point raised.*

The axis labels in the figures could be a bit clearer, maybe “estimated/expected”? Something that helps the reader quickly distinguish what the ratio is comparing.

*We thank the reviewer and have revised all figures and provided more complete descriptions of the axis.*

Additionally, I think the figure labels could better differentiate the comparison. For example, rather than “between/between” and “within/within”, why not “between/indirect” and “within/direct”? This may better orient the reader to the fact that it is a comparison of an estimate to the true population parameter.

It might help to provide some specific examples of when we might expect different scenarios of different combinations of direct and indirect effects (e.g., height vs educational attainment).

*We thank the reviewer for this helpful comment. We have provided some examples in the text*

I hope the authors take these comments in the constructive manner in which they are intended. This is a valuable analysis and important to those in the filed using polygenic scores
